# Cost analysis of eye bank versus surgeon prepared endothelial grafts

**DOI:** 10.1186/s12913-021-06828-z

**Published:** 2021-08-12

**Authors:** Luca Pagano, Kunal A Gadhvi, Mohit Parekh, Giulia Coco, Hannah J Levis, Diego Ponzin, Stefano Ferrari, Gianni Virgili, Stephen B Kaye, Rhiannon T Edwards, Vito Romano

**Affiliations:** 1 St. Paul’s Eye Unit, Royal Liverpool University Hospital, Liverpool, UK; 2 Department of Biomedical Sciences, Humanitas University, Via Rita Levi Montalcini 4, Pieve Emanuele, 20090 Milan, Italy; 3 UCL Institute of Ophthalmology, London, UK; 4 Department of Clinical Science and Translational Medicine, University of Rome Tor Vergata, Rome, Italy; 5 Department of Eye and Vision Science, Institute of Life Course and Medical Sciences, University of Liverpool, Liverpool, L7 8TX UK; 6 International Center for Ocular Physiopathology, The Venice Eye Bank Foundation, Venice, Italy; 7 Centre for Public Health, Queen’s University Belfast, Belfast, UK; 8 Department of Neurosciences, Psychology, Drug Research and Child Health (NEUROFARBA), University of Florence, Florence, Italy; 9 Department of Public Health, Institute of Population Health Sciences, University of Liverpool, Liverpool, UK; 10 Centre for Health Economics and Medicines Evaluation (CHEME), Bangor University, Bangor, UK; 11 Instituto Universitario Fernandez-Vega, Universidad de Oviedo and Fundacion de Investigacion Oftalmologica, Oviedo, Spain; 12 Department of Corneal and External Eye Diseases, St Paul’s Eye Unit, Royal Liverpool University Hospital, Prescot St, Liverpool, L7 8XP UK

**Keywords:** Cost, Corneal transplant, DSAEK, DMEK, Surgical expenses, Post-operative care expenses

## Abstract

**Background:**

Selective lamellar corneal transplantation (keratoplasty) has overtaken full thickness penetrating keratoplasty as the graft choice for endothelial failure. Even more recently eye bank prepared tissues are becoming increasing popular as a way to reduce the risks of tissue loss and stress during endothelial keratoplasty preparation in the surgical theatre. This study compares costs between surgeon and eye bank prepared tissues for Descemet’s stripping automated endothelial keratoplasty (DSAEK) and Descemet’s membrane endothelial keratoplasty (DMEK).

**Methods:**

Retrospective study conducted at the Royal Liverpool University Hospital including endothelial keratoplasties with a minimum of 6 months follow-up time. Cost analysis included surgical expenses, tissue acquisition fees, cost of patient’s ward admission and out-patient expenses, including cost of re-bubbling procedures, costs of visits, anterior segment imaging and optometrist visits within the first 6 months follow-up.

**Results:**

Ninety-eight eyes of 98 patients were included in the study of which 42 underwent DSAEK surgery and 56 DMEK surgery. Cost analysis of surgical expenses in the DSAEK group showed a significant difference between using surgeon prepared and eye bank prepared tissue (£3866 ± 296 and £4389 ± 360, respectively; *p* < 0.01) and the same was found in the DMEK group (£3682 ± 167 and £4162 ± 167 for surgeon prepared and eye bank prepared tissues, respectively; *p* < 0.01). Cost of out-patient visits did not differ significantly in either group.

**Conclusions:**

At the Royal Liverpool University Hospital, eye bank prepared tissues had higher surgical expenses compared to those prepared by the surgeon, while the post-operative care expenses were similar between the two groups.

## Background

Corneal transplantation is the most successful treatment in cases of advanced changes in corneal morphology or transparency. Over the past 20 years, the procedure for corneal transplantation has evolved from a full thickness graft (penetrating keratoplasty; PK) to replacement of only the effected corneal endothelial layer, called endothelial keratoplasty (EK). This is because of rapid rehabilitation, better ocular integrity and more predictable post-operative astigmatism after EK surgery [1–3]. In 2019, 30,650 EK procedures were performed in the United States of America alone, accounting for more than 60 % of the total grafts performed [4]. Similar trends are observed in Europe, with the most recent published annual European eye bank audit reporting 10,137 EK procedures compared to 8,169 PK emphasizing the shift towards EK [5, 6].

EK can be broadly divided into two procedures, the first being Descemet’s stripping automated endothelial keratoplasty (DSAEK), which is a relatively standardized and reproducible technique compared to its counterpart due to the use of a microkeratome to cut the graft tissue, thus making it a popular choice of surgery [7]. However, Descemet’s membrane endothelial keratoplasty (DMEK) represents the evolution of endothelial surgery into true lamellar surgery with only the dysfunctional endothelium being replaced by a healthy donor Descemet’s membrane and endothelium. Tissue preparation in DMEK has a higher risk of tissue loss, which is reported in 4.2-8 % of preparations [8, 9]. This association with a significant learning curve has led to increasing popularity of eye bank prepared pre-stripped and pre-loaded DMEK grafts to reduce the risks of tissue loss/damage and stress to the surgeon in the surgical theatre [10–13].

Both types of grafts can be prepared by the surgeon or by the eye bank technicians. Eye bank prepared tissues can save graft preparation time during surgery in addition to obtaining a fully validated tissue with regards to graft thickness for DSAEK and cell counts for both DSAEK and DMEK. Eye banks have access to spare tissue in the event of tissue loss due to preparation errors, which serves as an advantage over tissues prepared in the surgery, as loss of tissue in surgery leads to cancellation of surgery. There are several advantages associated with eye bank prepared tissues, such as the reduction in surgery time and instruments required in the surgery [14], however, it may lead to an increase in the tissue acquisition fee. Transplantation of a validated graft and reduced stress in surgery may compensate for the additional costs [15]. Although both, surgeon and eye bank prepared EK tissues are being widely accepted, there is little evidence evaluating the cost effectiveness of each approach [16], which is a crucial factor especially for small units with a lower number of transplants per year.

Different preservation media, preparation methods and transplantation options are used in different settings. In light of this, the aim of this study was to present a comparison of transplants using surgeon prepared and eye bank prepared tissues in terms of mean difference in cost per patient from an NHS England perspective, specifically, using the perspective of a large teaching hospital in the North West of the UK, The Royal Liverpool University Hospital (RLUH).

## Methods

In this retrospective study endothelial keratoplasty surgeries performed between January 2018 and August 2019 at the Royal Liverpool University Hospital (Liverpool, UK) with a minimum of 6 months follow-up time and with anterior segment optical coherence tomography (OCT) were included. The clinical study was approved by the Institutional Review Board (A0002786). We explored all direct medical costs for DSAEK and DMEK surgeries. Both DSAEK and DMEK surgeries were divided into surgeon cut (DSAEK) or surgeon stripped (DMEK) and eye bank prepared tissues (both pre-cut/stripped and pre-loaded). Eye bank prepared tissues (pre-loaded and pre-cut/stripped) were initially grouped together since the cost of tissue was the same, however, given the significant interest in pre-loaded, sub-group evaluation was also performed.

DSAEK surgeon prepared donor grafts were cut in theatre immediately before the transplant surgery. Donor corneas were mounted onto an artificial anterior chamber maintainer (Moria SA). Corneal epithelium was removed by using a polyvinyl alcohol sponge (Merocel, Alcon Laboratories, Inc.). Then, an automated microkeratome (Moria, SA) with a 350 mm head was used to remove the anterior corneal stroma. Manual dissection of the peripheral anterior stromal lamella was performed using a mini 1.25 mm crescent blade (Altomed, Ltd.) to avoid thicker peripheral graft edges. The remaining endothelium, Descemet membrane and approximately 100 μm of posterior corneal stroma was then punched using a Barron donor cornea punch (Hessberg-Barron, Katena Products, Inc.) at different diameters according to the surgeon’s choice for each patient.

DMEK surgeon prepared donor grafts were stripped in theatre immediately before the transplant surgery. The donor cornea was centered on a punch base using suction. The tissue was then stained with trypan blue for approximately 1 min to allow for better visualization. Then, either the stripping from trabecular meshwork method, or the scoring of the peripheral endothelium method was used, as described previously [17].

Eye bank prepared tissues all had quality checks before shipment with endothelial cell density (ECD) and thickness profile evaluation after preparation. DSAEK eye bank pre-cut tissues varied in diameter according to surgeon requests while DMEK pre-stripped tissues were cut at 9.5mm in diameter. The DMEK tissues were punched in theatre immediately before surgery with a Barron donor cornea punch (Hessberg-Barron, Katena Products, Inc.) to obtain the optimal size chosen for the specific patient. Pre-loaded grafts were shipped from the eye bank directly inside the injector, in a ready-to-use fashion.

We measured resource use for all medical and surgical related care along with follow-up outpatient care over a six months period. Surgical resource use was defined as the cost associated at the time of surgery and costs related to patients’ hospital admission. They included tissue acquisition fees, cost of surgery and cost of ward admission.

Out-patient resource use was defined as all eye health related service contacts and related costs occurring after surgery for a six month follow up period. They included cost of re-bubbling procedures, cost of out-patient visits, cost of anterior segment imaging (anterior segment OCT; AS-OCT) and cost of optometrist visits. The 6-month post-operative timepoint was chosen since most EK complications are known to occur over the first 6 months after surgery. Tissue acquisition fees, depending on the type of tissue received (tissue for PK for surgeon prepared tissue or pre-cut, pre-stripped or pre-loaded tissue if prepared by the eye bank) was obtained from The Venice Eye Bank Foundation.

There are several different methods for costing hospital contacts. Choice of any method needs to be justified. We took a low costing method as this was a single-site study. Costs for ward admission, out-patient visits, optometrist visits and anterior segment imaging were provided by the Royal Liverpool University hospital. Costs of EK surgeries and re-bubbling procedures were obtained from the National NHS tariff [4]. This was a single site retrospective costing and cohort study combining costing data from the finance department with NHS tariff costing information. We did not use NHS reference costs, which could have been an alternative approach, particularly if this had been a multi-site study. Even using national reference costs, outcomes can be different depending on which ones are used [18].

Data on patients’ diagnosis, best corrected visual acuity (BCVA) pre- and post-operatively and surgeon graft preparation failure were also collected. All costs were for in pound sterling and reflect the price for 2018–2019, which was the same.

Th main outcome was to evaluate the presence of a between-group difference in mean treatment cost in EKs between surgeon prepared and eye-bank prepared tissues both at the time of surgery and in the post-operative period up until 6 months after surgery from an NHS perspective, specifically, using the perspective of a large teaching hospital in the North West of the UK (RLUH).

The statistical analyses were performed using STATA 14.0 (StataCorp, College Station, TX). The normality of all the data was estimated using the Shapiro-Wilk normality test. Since data were not normally distributed, comparisons between surgeon prepared and eye bank prepared tissues for DSAEK and DMEK were performed with the two-sample Wilcoxon rank-sum test (Mann-Whitney). A p-value less than 0.05 was set for statistical significance. Results are presented as mean ± standard deviation and interquartile range for continuous variable and percentages for categorical variables.

## Results

A total of 98 consecutive patients (98 eyes) undergoing DSAEK or DMEK surgery in our tertiary referral centre between January 2018 and August 2019 were included after surgeon cut DSAEK (*n* = 10), eye-bank prepared DSAEK (*n* = 32), surgeon cut DMEK (*n* = 28) and eye-bank prepared DMEK (*n* = 28). No tissue wastage was noted in the surgeon prepared group. In the DSAEK group, 25 eyes (60 %) had Fuchs’ endothelial corneal dystrophy (FECD) and 17 (40 %) had bullous keratopathy. In the DMEK group 52 (93 %) had FECD and 4 (7 %) had bullous keratopathy. The mean age of treated patients was 72.4 ± 10 years and 46 % were male. Mean corrected distance visual acuity improved from 0.97 ± 0.7 LogMAR preoperatively to 0.37 ± 0.44 LogMAR 6 months postoperatively (Table 1).

**Table 1:** Characteristics of our patient cohort

Baseline characteristics	
Number	98
Age	72.4 ± 10 [38, 90]
Sex	45 M, 53 F
Reason for graft	77 Fuchs’ Endothelial corneal dystrophy
	21 Bullous Keratopathy

Age as mean ± standard deviation. [minimum, maximum], sex is total number of each male and female

**Fig. 1:**
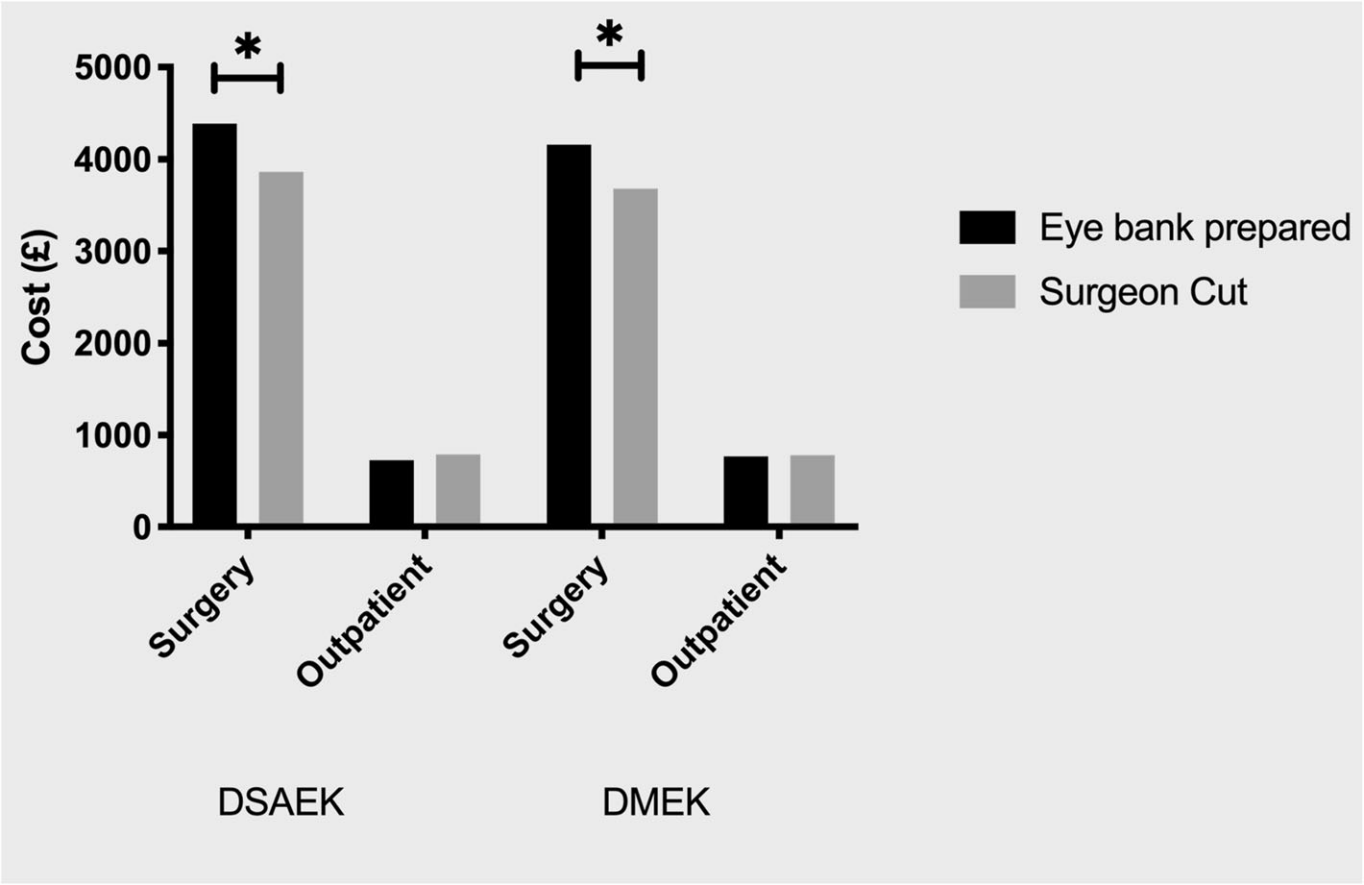
Graph showing the cost (in £) for different procedures for the surgery and the outpatient department

The detailed breakdown of costs and acquisition fees for each type of graft are shown in Table 2. The eye bank prepared grafts were associated with higher costs (4389 ± 360, IQR: 4312;4312, min 3699, max 5538) compared to the surgeon prepared grafts (3866 ± 296, IQR: 3682;4295, min 3682, max 4295) in cases of DSAEK (*p* < 0.01) and DMEK, which were 4162 ± 167 (IQR: 4162;4162, min 3549, max 4775) and 3682 ± 167 (IQR: 3682;3682, min 3069, max 4285), in eye bank prepared and surgeon prepared, respectively (*p* < 0.01; Fig. 1). This difference, however, might be partially compensated for by the time saved in the theatre not having to prepare the tissues, resulting in a faster surgical time [19].

**Table 2:** Detailed summary of the cost of every aspect of surgical and outpatient management in the first 6 months after the operation. Numbers represent the average total cost per patient of each specific activity and are expressed in £ ± standard deviation. In square brackets there are the interquantile ranges. Numbers in round brackets represents the average number of units per group. E.g.in the DSAEK surgeon group, on average we performed 2.6 OCT scans for each patient, with a cost per scan of £67 (average total cost per patient: £67 x 2.6 = £174).

Resources	Unit	Unit cost (£)				Details
		DSAEK Surgeon	DSAEK Bank	DMEK Surgeon	DMEK Bank	
**Surgery costs**						
Graft	Tissue	1640	2251	1640	2120	Cost of tissue
Surgery	Procedure	1429	1429	1429	1429	Cost of procedure
Ward	Day	797 ± 296 [IQR: 613;1226] (1.3)	709 ± 351 [IQR: 613;613] (1.15)	613 ± 167 [613;613] (1)	613 ± 167 [613;613] (1)	Cost per day of admission: £613
Total		3866 ± 296 [IQR: 3682;4295]	4389 ± 360 [IQR: 4312;4312]	3682 ± 167 [IQR: 3682;3682]	4162 ± 167 [IQR: 4162;4162]	
*p*-value		<0.01		<0.01		
**Outpatient costs**						
Rebubbling	Procedure	0 ± 0 [IQR: 0;0] (0)	50 ± 134 [IQR: 0;0] (0.13)	14 ± 76 [IQR: 0;0] (0.04)	0 ± 0 [IQR: 0;0] (0)	Cost of RB in theatre: £400
Follow up	Visit	536 ± 133 [IQR: 447;626] (6)	486 ± 136 [IQR: 447;581] (5.4)	517 ± 142 [IQR: 447;626] (5.8)	511 ± 143 [IQR: 357;626] (5.7)	Cost of visit: £89.40
Optometrist	Visit	79 ± 83 [IQR: 0;158] (0.5)	74 ±106 [IQR: 0;158] (0.5)	85 ± 91 [IQR: 0;158] (0.5)	51 ± 87 [IQR: 0;158] (0.3)	Cost per visit: £158
OCT	Imaging	174 ± 123 [IQR: 67; 268] (2.6)	117 ± 110 [IQR: 34; 201] (1.7)	165 ± 104 [IQR: 67; 201] (2.5)	208 ± 136 [IQR: 134; 335] (3.1)	Cost per scan: £67
Total		789 ± 226 [IQR: 693;871]	727 ± 322 [IQR: 503;884]	781 ± 273 [IQR: 593;917]	769 ± 276 [IQR: 536;961]	
*p*-value		0.78		0.99

Outpatient costs of DSAEK and DMEK grafts, show that there is no difference (p = 0.78 and 0.99, respectively) if prepared by surgeon or by the eye bank in the first 6 months of follow up, when there are usually more interventions.

## Discussion

In our cost analysis study of endothelial keratoplasty we identified that surgeries using eye bank prepared grafts resulted in a significantly higher cost per patient compared with EK using surgeon-prepared grafts in both DSAEK (*p* < 0.01) and DMEK (*p* < 0.01) from an NHS England perspective, specifically, using the perspective of a large teaching hospital in the North West of the UK. We used costs from RLUH as well as tariff costs for England as a whole. These costs were not weighted by the market forces factor as for Royal Liverpool University Hospital this was close to 1 (NHS England, 2014 [20]). In particular, we found these increased expenses were mainly due to higher surgical expenses, while no differences were found in expenses related to the post-operative care of both groups.

The choice between surgeon and eye bank prepared grafts should not only be based on the cost. DSAEK has become a more standardized procedure since the advent of automated modern keratome techniques [7], however, lenticules prepared by surgeons still lack validation. This validation is particularly important since recent evidence supports a trend towards ultra-thin DSAEK lenticules, which demonstrate a better visual outcome following surgery [21, 22] less hyperopic shift [23] and higher order aberrations [24]. However, preparing and validating tissue requires infrastructure and time, including access to accurate pachymetry, which is unavailable intraoperatively to most surgeons.

Similarly, many methods for DMEK tissue preparation have been described including the no touch stripping technique, pneumatic dissection, hydro-separation, and others [25–29]. Many of these methods are not validated in the hands of novice DMEK surgeons and, consequently, the quality of tissues prepared may vary compared to results from standardized techniques [30]. During the learning curve, tissue loss can vary significantly and is reported in approximately 4.2-8 % of preparations [9, 8]. This may result in additional costs and cancelled surgeries and, most importantly, loss of precious human donor tissue which is in short supply. However, with refinement and experience, the proportion of tissue losses can drop to 1 % [31] together with a reduction in the endothelial cell loss during preparation [13]. Nevertheless, questions regarding DMEK graft validation persist, particularly in regard to endothelial cell loss during surgeon preparation. This may support the use of eye bank prepared controlled and validated tissue regardless of their higher costs [32].

Although there was no reported tissue loss in our study during preparation of the DMEK or DSAEK grafts by surgeons, it is difficult to speculate on endothelial cell counts or thickness profile. This is largely due to the fact that surgical theatres are not equipped with instruments like OCT and inverted or specular microscopes that can obtain this information.

Furthermore, eye bank-prepared tissues resulted in an initial increase in costs, this difference can partially be compensated by the time saved for tissue preparation in theatre, resulting in shorter theatre time usage [19], thus giving the chance to accommodate more surgeries in the same day[19]. Busin et al. demonstrated a potential increase in efficiency using 46 DMEK grafts[10]. The study reported an average preparation time in the eye bank of 26 min, however the time in theatre from opening the graft until anterior chamber air injection could be consistently reduced, potentially freeing up theatre space [10, 31]. Unfortunately, since theatre time was not measured in this study, we were unable to quantify the amount of time saved and to attribute a specific monetary value to this.

This was a partial economic evaluation at group level based on a retrospective cohort [33–35]. However, we argue here that our paper could inform a full trial and full economic evaluation and moves the field on in terms of choice of methods of surgery and post-operative care. Evaluation of these costs are also useful to tissue engineers working in the field of corneal transplantation as it could contribute to a health economics study assessing the potential market for a tissue engineered graft.

## Conclusions

We present a between-group cost analysis alongside a retrospective cohort study of DSAEK and DMEK surgeon prepared and eye bank prepared grafts over the first 6 months after surgery. We found that EK using eye bank prepared grafts were more expensive than surgeon prepared grafts at the Royal Liverpool University Hopsital. This difference was only evident in the total surgery cost and mainly linked to the higher acquisition fee of eye bank-prepared tissues. However, these increased costs need to be balanced with advantages of shorter theatre time usage and the availability of tissues with documented quality control.

## Data Availability

The datasets used and/or analysed during the current study are available from the corresponding author on reasonable request.

## Abbreviations

AS-OCT: Anterior segment optical coherence tomography
BCVA: Best corrected visual acuity
DMEK: Descemet Membrane Endothelial Keratoplasty
DSAEK: Descemet’s stripping automated endothelial keratoplasty
EK: Endothelial keratoplasty
ECD: Endothelial cell density
FECD: Fuchs’ endothelial corneal dystrophy
PK: Penetrating Keratoplasty
NHS: National Health Service
